# The Effect of Diet on the Composition and Stability of Proteins Secreted by Honey Bees in Honey

**DOI:** 10.3390/insects10090282

**Published:** 2019-09-02

**Authors:** Oleg Lewkowski, Carmen I. Mureșan, Dirk Dobritzsch, Matthew Fuszard, Silvio Erler

**Affiliations:** 1 Institut für Biologie, Molekulare Ökologie, Martin-Luther-Universität Halle-Wittenberg, Hoher Weg 8, 06120 Halle (Saale), Germany; 2 Institutul de Științele Vieții “Regele Mihai I al României”, Nutriție moleculară (Genomică și Proteomică), Universitatea de Științe Agricole și Medicină Veterinară, Calea Mănăștur 3-5, 400372 Cluj-Napoca, Romania; 3 Proteinzentrum Charles Tanford, Core Facility-Proteomic Mass Spectrometry, Martin-Luther-Universität Halle-Wittenberg, Kurt-Mothes-Straße 3a, 06120 Halle (Saale), Germany; 4 Institut für Biochemie und Biotechnologie, Pflanzenbiochemie, Martin-Luther-Universität Halle-Wittenberg, Kurt-Mothes-Straße 3a, 06120 Halle (Saale), Germany; 5 Zentrum für Medizinische Grundlagenforschung (ZMG), Medizinische Fakultät der Martin-Luther-Universität Halle-Wittenberg, Ernst-Grube-Str. 40, 06120 Halle (Saale), Germany

**Keywords:** *Apis mellifera*, major royal jelly proteins, invertase, diastase, glucose oxidase, honey production, honey ripening, mass spectrometry

## Abstract

Honey proteins are essential bee nutrients and antimicrobials that protect honey from microbial spoilage. The majority of the honey proteome includes bee-secreted peptides and proteins, produced in specialised glands; however, bees need to forage actively for nitrogen sources and other basic elements of protein synthesis. Nectar and pollen of different origins can vary significantly in their nutritional composition and other compounds such as plant secondary metabolites. Worker bees producing and ripening honey from nectar might therefore need to adjust protein secretions depending on the quality and specific contents of the starting material. Here, we assessed the impact of different food sources (sugar solutions with different additives) on honey proteome composition and stability, using controlled cage experiments. Honey-like products generated from sugar solution with or without additional protein, or plant secondary metabolites, differed neither in protein quality nor in protein quantity among samples. Storage for 4 weeks prevented protein degradation in most cases, without differences between food sources. The honey-like product proteome included several major royal jelly proteins, alpha-glucosidase and glucose oxidase. As none of the feeding regimes resulted in different protein profiles, we can conclude that worker bees may secrete a constant amount of each bee-specific protein into honey to preserve this highly valuable hive product.

## 1. Introduction

Honey, the carbohydrate source for honey bee colonies, is produced by in-hive worker bees through a process of ripening foraged nectar, honeydew or other sweet plant saps (e.g., inversion of sugar) until long-storable honey is obtained [1,2]. Carbohydrates (mainly glucose and fructose), minerals, amino acids, plant secondary metabolites and proteins can be found in variable amounts, each being characteristic of specific honey types. Proteins detectable in honey (0.58–7.86% [3]) are mainly secreted from salivary and hypopharyngeal glands of forager and in-hive bees [4] and might be of minor relevance for larval and adult bee nutrition. Actually, the nutritive value of honey proteins is not clear and needs to be investigated in future studies, and additionally, alternative health-enhancing and developmental functions of honey proteins for larvae and adult bees are possible. Furthermore, it is known that pollen is the main protein and amino acid source for bees to facilitate gland development and brood rearing. 

Two major groups of bee-secreted proteins are omnipresent in all types of honey: (1) carbohydrate metabolism enzymes and (2) royal jelly characteristic proteins. The first group includes glucose oxidase (generates antimicrobial H_2_O_2_ from glucose) [5], invertase (digestion of sucrose to glucose and fructose—also known as alpha-glucosidase III [6], saccharase, sucrase) and diastase (active enzymes: alpha-, beta-amylase) [7]. These bee-secreted enzymes are responsible for the transformation of honey’s chemical composition; in particular, the sugar spectrum. They are mandatory for the conversion of polysaccharides to di- and monosaccharide, which are essential energy sources. Enzyme activity can already be detected in the honey stomach of bees exclusively fed with sugar solution [8], which shows that enzymatic activity is independent of floral origin [8]. Glucose oxidase, for instance, is constitutively expressed and secreted by nurse bees into larval food jelly and by foragers into honey [9]. The second group of proteins contains major royal jelly proteins 1–5 (mostly MRJP1 [10]), royalisin (known as defensin-1 [11]) and apisimin [12]. These proteins are important for honey’s antimicrobial activity and are the most abundant proteins of the total protein quantity [13]. The antimicrobial activity of MRJPs, combined with substances (e.g., H_2_O_2_, gluconic acid) generated during sugar conversion, are thought to prevent honey from microbial spoilage. More details on the identification and characterization of bee-produced honey proteins can be found in Chua et al. [4].

Recently, proteins isolated from mono- and polyfloral honeys were tested as potential markers of the geographical and botanical origin of honey [13,14]; for example, via the fingerprinting and barcoding of proteins using mass spectrometry [15]. Honey proteins not only differ for honeys produced from different plant sources, but the spectrum also differs significantly between bee species foraging on the same floral source (e.g., *Apis mellifera* vs. *Apis cerana* [16]). Floral proteins are rarely detected in honey protein profiles (except for buckwheat, eucalyptus, sunflower and canola honey; Figure S1 and Table S1 [14,17]) and usually make up a small fraction of total protein content. Minor quantities of plant proteins might be digested by honey bee proteases [18], filtered (pollen particles) or degraded during honey ripening [14,19]. All studies using honey proteins as markers of freshness, quality, degree of adulteration (extension with sugar, syrup or water), or simply to characterize monofloral honeys have applied several methods (1- or 2-dimensional sodium dodecyl sulfate polyacrylamide gel electrophoresis (SDS-PAGE), gel-free based analysis including mass-spectrometry), resulting in a highly variable quality and quantity [17,20]. 

Honey protein composition and quantity have been the main topics studied in the last few decades, without a strong focus on their biological relevance for the honey bee itself. In this study, we focus on investigating nutritional factors influencing both protein composition and quantity in the process of honey ripening. We address the question of whether food-processing bees are sensitive to different food compositions and therefore respectively adjust their protein spectra and quantity dependent on the supplied carbohydrate source to guarantee a constantly high quality of honey proteins, in particular as an antimicrobial source for the colony. Hive bees have different food sources in their colony (e.g., honey, fresh pollen, bee bread) with a very heterogeneous composition (e.g., carbohydrates, amino acids, plant secondary metabolites), all of which may influence the quality and quantity of honey protein profiles. To avoid effects of external factors, such as climate, geography (soil, minerals, humidity) and botanical (plant genotype, different floral sources) or biological factors (brood, queen, contamination by the colony), controlled cage experiments were limited to observations resulting from different sugar sources and several additives. Furthermore, a time series was used to measure short-term protein stability (potential degradation) directly upon storage in cells of the honey comb.

## 2. Materials and Methods

### 2.1. Bee Feeding Regime; Honey Ripening, Storage, and Protein Analysis

*Apis mellifera carnica* worker honey bees were collected from honey frames (performing honey processing tasks) to avoid the sampling of freshly emerged, forager, wax bees and guardians. For the experiment, bees were housed in wooden cages (13 cm × 11 cm × 8 cm, in groups of approximately 40 bees) with pieces of freshly prepared empty combs (from the same hives) attached to the cage wall [21]. The bees were starved for one hour and then supplied with different nutrients ad libitum—pure multifloral honey or 50% sucrose solution (w/w)—to compare highly complex hive food with simple sugar solution. After 3 days, honey-like product samples were collected carefully from single cells of the honey comb using a pipette and stored at −20 °C until protein profile analysis. Previous experiments already showed that providing honey bee colonies with sucrose solution (>40% w/w) resulted within 3 days in a product with a total sugar concentration above 80%, which is the value of ripened honey [22,23]. To verify the honey-like character of the stored products from this study, sugar concentrations of three random samples from all feeding regimes were determined using a refractometer.

**Figure 1:**
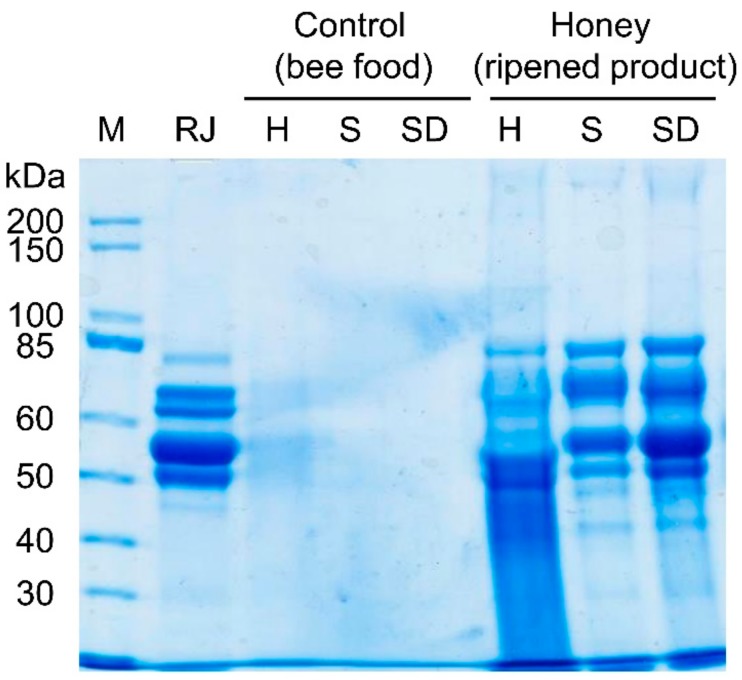
Eight percent sodium dodecyl sulfate polyacrylamide (SDS PA) gel showing protein profiles of bee food (H: honey (diluted 1:1 with ddH_2_O), S: 50% sucrose, SD: 50% sucrose + blue dye) and resulting honey-like products, extracted from three-day-old products. Royal jelly protein extract (RJ) was used as a control, as most proteins detected in honey are major royal jelly proteins. (M: marker; unstained protein standard, broad range (10–200 kDa) (New England Biolabs, Ipswich, MA, USA)).

Following initial screening (Figure 1), sugar solution with different additives was shown to be the most suitable nourishment to study the impact of different food sources on honey proteome composition and stability. Four different feeding regimes (ad libitum) were selected (each with three replicates): (1) 50% sucrose solution (w/w) only; (2) 50% sucrose solution (w/w) and polyfloral pollen in addition (origin: Romania), ad libitum; (3) 50% sucrose solution (w/w) plus quercetin (2.26%, w/v); and (4) Apiinvert^®^ (common artificial bee food; mix of 31% sucrose, 30% glucose and 39% fructose) only, diluted to 50% total carbohydrate concentration. The latter diet served as a control for the combination of mono- and di-saccharides. Quercetin is a major plant secondary metabolite (flavonoid) frequently found in nectar, pollen and bee products [24,25] and is a significant and attractive cue of numerous plant nectar sources for honey bee foragers [26]. We used a concentration that clearly exceeds the usual concentration found in nectar in the field to assure the perception of the flavonoid from the sugar solution and trigger a potential response of worker bees. To follow honey production and ripening on honey combs, all food solutions were stained with 0.1% Brilliant Blue FCF (Figure S2) according to Ehrenberg et al. [27]. Staining sugar solutions were shown not to influence the honey-like product protein composition (Figure 1). A few days after setting up cages, a minimum of two samples (which means two individual cells) of each cage (three cages per feeding regime) were analysed by gel electrophoresis. 

**Figure 2:**
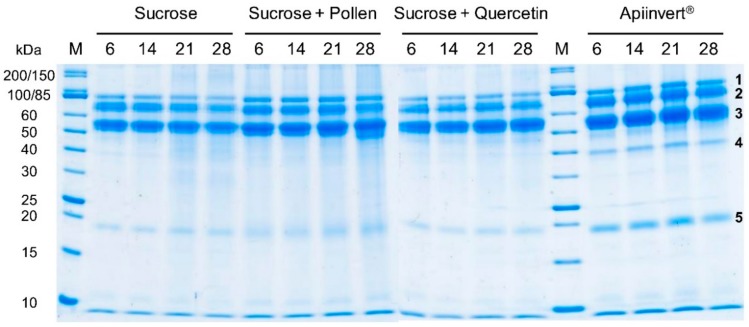
Twelve percent SDS PA gels showing the temporal dynamics of honey-like product protein profiles based on different feeding regimes. Bold numbers (1–5) indicate the five bands used for density quantification. (M: marker, unstained protein standard, broad range (10–200 kDa) (New England Biolabs, USA); 6–28: storage time in days).

Changes in the protein stability of randomly picked, freshly ripened honey-like product samples were studied while storing them for 28 days at 35 °C. Samples of each treatment group were taken once a week (day 6, 14, 21 and 28). The honey proteome composition and stability was measured using sodium dodecyl sulfate polyacrylamide gel electrophoresis (SDS PAGE; 12% gels, Colloidal Coomassie staining–Brilliant Blue G-250), gel imaging (gels scanned with 600 dpi, auto white balance, 24 bit depth, RGB colour representation, and captured with ImageJ 1.48v) and software analysis (GelAnalyzer 2010a) as described in the work by Mureșan and colleagues [28]. Before loading and running gels, samples were diluted to 1:1 with distilled water, and 10 µL of each sample was incubated with 5 µL 5 × SDS Laemmli sample buffer [28] at 95 °C for 5 min (standard gels) or at 70 ℃ for 10 min (protein stability gels). Electrophoresis was performed at a constant voltage of 175 V for 1 h (standard gels). Protein stability gels were run at 80 V for 1 h and afterwards 175 V for 1 h to increase resolution. GelAnalyzer was used to estimate raw volume for the five most common protein bands (Figure 2) of each sample (5–6 replicates per treatment group) [28]. To account for variance in total protein amount, 4 of the 5 protein bands are given as relative values, normalized to the band with the highest density (always the band at 50–60 kDa, Figure 2). 

### 2.2. Protein Precipitation, Dye Removal, and Identification via Mass-Spectrometry

Fifty microliters of dyed honey-like product was diluted with distilled water (up to 500 µL final volume); proteins were precipitated using 1% NaDOC (50 µL) and 50% TCA (110 µL) and incubated for 30 min on ice. After centrifugation (~18,000× *g*, 10 min), the supernatant was removed and the pellet washed in 200 µL ice-cold acetone. Following another identical washing step, the protein pellet was air-dried and washed five times in 500 µL Tris-HCl (0.1 M, pH 8.0) to remove most of the blue dye. Next, this light blue-coloured pellet was washed four times in 500 µL ammonium bicarbonate (25 mM), including incubation at 37 °C for 1 h. In the last washing step, the protein pellet was incubated at 37 °C, overnight, in 500 µL DMSO under shaking conditions (300 rpm). After the effective removal of the blue dye, samples were centrifuged for 10 min (~18,000× *g*), and DMSO was removed and prepared for mass-spectrometry.

Mass spectrometric analyses were performed using the principles described earlier [29]. Briefly, 1 µL of tryptic peptides (~400 ng peptides) was trapped on a 20 mm × 180 µm fused silica M-Class C18 trap column (Waters, Eschborn, Germany) and washed for 5 min at 5 µL/min with a 1% solution of 0.1% formic acid (FA) in acetonitrile (ACN) in 99% trifluoroacetic acid (0.1% in water) before being separated on a 250 mm × 75 µm fused silica M-Class HSS T3 C18 column (with 1.8 µm particle size) (Waters, Eschborn, Germany) over a 35 min gradient consisting of increasing concentrations of 7–40% of 0.1% FA in ACN within 0.1% FA in water (Carl Roth, Karlsruhe, Germany). Eluting peptides were ionized at 2.1 kV from a pre-cut PicoTip Emitter (New Objective, Woburn, MA, USA) with source settings of 80 C and a nano N_2_ flow of 0.4 bar. Ions passed into the Synapt G2S Mass Spectrometer (Waters, Eschborn, Germany), which was operated in both the positive ion mode and resolution mode with the following settings: ion trap cell mobility separation with a release time of 500 μs, and afterwards “cooled” for 1000 μs; the helium pressure was set to 4.7 mbar, and the IMS cell nitrogen pressure was 2.87 mbar; the wave height was 38 V, and the wave velocity was ramped from 1200–400 m/s. Glu-1-Fibrinopeptide B (250 fmol/μL, 0.3 μL/min) was used as lock mass (*m/z* = 785.8426, z = 2).

The RAW MS mzML data files were generated with ProteinLynx Global Server (PLGS) 3.0.1 (Waters, Milford, MA, USA) with the following settings: automatic calculation of chromatographic peak width and MS TOF resolution, the lock mass for charge 2 was ‘785.8426 Da/e’, thresholds were set to 135 counts for low energy, 80 counts for elevated energy and 750 counts for intensity, respectively. These mzML files were initially run through the sampling search engine Preview v3.3.11 (Protein Metrics, Cupertino, CA, USA) against *A. mellifera* (Amel_4.5, https://www.ncbi.nlm.nih.gov/assembly/GCA_000002195.1), which generated full search parameters: a precursor mass tolerance of 20 ppm and a fragment mass tolerance of 30 ppm. Digestion specificity was set to trypsin with possible N-raggedness. The lock mass Glu-1-Fibrinopeptide B was used for the recalibration of fragments and precursors. A database search was set with these modifications: the fixed modification of carbamidomethylation on Cys, variable modifications of (di) oxidation on Met, N-terminal Gln->pyro-Glu and Glu->pyro-Glu conversion, N-terminal acetylation, and deamidation of Gln. Full searches were run through Byonic v3.3.11 (Protein Metrics, Cupertino, CA, USA) with the additional following settings: the maximum number of precursors per MS2 was set to 10, as recommended by the manufacturer for MSE data. Protein FDR was set to 1%, against both *A. mellifera* (Amel_4.5) and an internally curated focused database generated for *A. mellifera* hypopharyngeal glands and brains, consisting of sequences emphasised for uniqueness (database curation procedure published earlier [30]). Output mzIdentML files were then loaded into Scaffold Q + S 4.8.9 (Proteome Software Inc., Portland, OR, USA).

## 3. Results

All honey-like products analysed in this study were shown to have increased sugar concentrations (sucrose solution only: 57–74%, sucrose solution plus pollen: 65–68%, sucrose solution plus quercetin: 57–76%, Apiinvert: 73–77%) in comparison to 50% sugar solution (starting material). On the other hand, all sugar values were lower than for ripened honey (some random samples: 80.5–82.5%). This means that all products are worker bee-processed sugar solutions, which are on their way to becoming ripened honey.

Comparing the protein profiles of honey-like products produced by honey-processing worker bees fed with sucrose solution versus pure polyfloral honey indicated that sucrose solution-based honey-like products showed the typical protein bands known from many different honey types, with clearly identifiable bands between 45 to 85 kDa (Figure 1 and Figure S1; in accordance with [13]). The band with the highest density is always a product at 50–60 kDa (Figure 1 and Figure 2). Royal jelly protein extract (see [28] for details) showed a comparable protein profile, which was the same as in pure sucrose-based honey-like product protein samples. The royal jelly extract was used as the positive control, since it is known that honey resembles royal jelly in protein composition [19]. Multifloral honey-fed worker bees produced honey-like products with a blurry, non-typical protein profile (Figure 1), caused by modified proteins, as a result of processes such as glycation (see Section 4 for details).

High-resolution denaturating PA gels (12%) revealed additional protein bands between 15–20 kDa as well as around 10 kDa for all four treatment groups (Figure 2). The statistical comparison of protein band densities for the most common bands demonstrated that the protein concentrations differ significantly from each other (Kruskal-Wallis ANOVA, H = 70.64, dF = 3, n = 84, *p* < 0.0001); however, the honey-like products of the four treatment groups do not (K-W ANOVA: H = 2.15, dF = 3, n = 84, *p* = 0.54) (Figure S3). This means that neither the simultaneous ad libitum feeding of floral proteins (pollen) nor the addition of a plant secondary metabolite (quercetin) influence the protein composition and quantity of bee-secreted honey proteins. This was also valid for a more complex sugar food source (Apiinvert) compared to sucrose only (Figure S3).

The temporal analysis of honey-like product protein profiles showed no remarkable change (Figure 2). Consequently, the short-term storage (up to 4 weeks) of freshly ripened honey-like products in this study did not cause a major detectable degradation of proteins added by worker bees. However, the stored product from sucrose solution plus pollen feeding, on day 28, showed that slight signs of degradation may have occurred (Figure 2). As the major band (~55 kDa, MRJP1) remained unchanged and the lower product (~50–55 kDa) became shorter, MRJP2 is the candidate for the shortened product. For the Apiinvert protein dynamics, a single product ~40 kDa seems to be unique for this honey-like product; however, comparing different samples of all four treatment groups showed that this product is also present in the other three honey-like products (Figure S4). Differences seen in Figure 2 might originate from the normal variance in protein staining. 

Complete honey-like product proteome mass spectrometry analysis confirmed that honey bees, irrespective of their diet, add seven different proteins to ‘nectar’ (sugar solution) in the process of honey ripening. The proteins are produced in the hypopharyngeal glands, including MRJP1, 2, 3, 5 and 7, alpha-glucosidase (Hbg3) and glucose oxidase (Table 1). The relative abundance of each protein per treatment group did not differ between feeding regimes (Χ^2^-test: Χ^2^ = 4.99, *p* = 0.96). This implies that the addition of food additives does not result in differences in protein quantity and composition. The majority of all honey proteins were MRJP 1, 2 and 3, with MRJP1 being the most abundant and MRJP2 and 3 being detected in equal concentrations (Table 1). In spite of the very high similarity of MRJPs [31], a distinct identification of MRJP 5 and 7 was possible, using the modified reference honey bee protein data base [30] that excluded similar peptides of highly related proteins. The molecular weight (MW) of all identified proteins matched well with the protein bands shown in Figure 2 (MW values see Table 1). Using modified identification criteria, the protein band below 20 kDa (Figure 2) might be a protein identified with a molecular weight of ~19.5 kDa; however, this is of unknown function (XP_397512.1, uncharacterized protein LOC408608-*Apis mellifera*). This protein has also been identified in monofloral honey samples using classical protein identification (mass spectrometry of proteins cut out from SDS gels) (Table S1).

## 4. Discussion

Sugar solution-based honey-like products include exclusively bee-specific proteins. This is in accordance with previous studies [8,32]. The investment of worker honey bees adding proteins based on the individuals’ reserves is not without purpose: storing bee-produced proteins in honey(-like products) prevents or at least strongly decelerates protein degradation (Figure 2) and therefore provides an alternative protein storage strategy compared to direct storage in secreting gland tissue (even by overwintering bees [33]) or haemolymph. Long-term experiments have shown that honey protein content decreases by 46.7% after 6 months, independent of the botanical origin [34]. However, under natural conditions, honey might not be stored for longer than 6 months in the hive, as honey bees produce and consume honey regularly, depending upon the brood status, number of individuals and flowering season. The general energy requirements of the colony for processes such as temperature regulation are also a critical factor influencing honey storage duration [35,36]. Furthermore, the storage of honey in wax cells and finally cell capping may significantly contribute to counteracting—and/or the retardation of—the decay of proteins and other honey compounds. 

Major royal jelly proteins, alpha-glucosidase and glucose oxidase dominated the spectral counts with protein profiles of the different honey-like products, independent of the food source (with or without additives) (Figure 2, Table 1). Thus, as mentioned earlier, honey proteomes resemble the royal jelly proteome [19], which has, in addition, enzymes relevant for honey bees’ carbohydrate metabolism. Alpha-amylase was not detected in any of the samples. This is expected, as this enzyme (~56 kDa) is secreted into honey exclusively by forager bees [37], which was precluded by the experimental design. Worker bees may need an environmental or another signal (perceived while foraging for nectar and pollen) to activate the secretion of amylase, which is required to convert starch of plant origin (mainly from pollen) into maltose. Furthermore, we were unable to detect apisimin (~8 kDa) and defensin-1 (~11 kDa). This is unsurprising, as apisimin generates only one possible tryptic peptide which may not ‘fly’ in mass spectrometric analyses [30], and defensin-1 is mostly present in honey with <1% of the total honey proteome [38], which might explain its absence in our samples. Nevertheless, in Figure 2, a single band (evenly present in all samples) slightly above 10 kDa can be observed, which might be defensin-1 (10.717 kDa). Specific antibodies targeting apisimin or defensin-1 [11,38] need to be used in future studies to confirm the presence of both proteins. 

Protein sizes, band clearness and brightness on SDS gels varied between tested hive products (royal jelly proteins isolated from royal jelly versus those isolated from honey-like products) and the different bee foods initially tested (honey versus sugar solution) (Figure 1, Figure 2, Figure S1 and Figure S4). This observation is based on protein modifications caused by nectar polyphenols and carbohydrates [34,39], known as glycation. In contrast to the well-known glycosylation (*N*- or *O*-linked oligosaccharides as result of enzymatic reactions), glycation is based on an enzyme-independent chemical modification known as Maillard reaction. These modifications lead to slightly higher molecular weight proteins compared to unmodified proteins (an increase of 3–5% of total molecular weight [40]) and therefore demonstrate a slower migration behaviour of proteins on an SDS PAGE gel, leading to bands riding higher on the gel. 

The successful identification of honey proteome composition not only depends on the resolution and sensitivity of the used analytical method, but also on a careful and clean sampling process. Using a state-of-the-art technique, Erban and colleagues [18] failed to detect profilin, superoxide dismutase and apisimin, which are known as honey proteins [12]. On the other hand, they were the first to describe many thus-far undiscovered honey proteins (e.g., hymenoptaecin, venom-related and venom-like proteins, proven allergens, serine proteases, inhibitors of serine proteases, and isoforms of glucose dehydrogenase). Our study also failed to detect the rare honey proteins described in both studies [12,18]. This could be the result of methodological issues (extraction efficiency, detection sensitivity, etc.) or simply a lack of contamination. Honey bee-driven contamination from honey bee proteins not supplied to honey, by workers, is discussed as a major reason why Erban and colleagues detected so many unknown honey proteins [18]. These newly-detected proteins might belong to larvae remaining in the combs or bee venom, used by worker bees for comb disinfection [24,41], at the time of honey extraction [18]. As a consequence, future studies should use freshly prepared honey frames and extract honey from single cells or specific parts of the comb to avoid interference from cross-contamination. 

Floral proteins, stored in-hive as pollen and bee bread, are the major nitrogen source of adult honey bees. Larval honey bees mainly rely on royal jelly as a protein source. Proteins identified in honey, including MRJPs, may contribute to the health and development of larvae and adult honey bees or present an alternative nitrogen resource for the whole colony. However, total quantities of proteins are lower for honey-based proteins in comparison to pollen, bee bread or royal jelly. Consequently, it may not only the amount of honey proteins but also their chemical modifications (e.g., glycation and glycosylation) that make them indispensable for still-unknown biological functions. Currently, the enzymatic (carbohydrate metabolism) and antimicrobial activity (e.g., apisimin, defensin, MRJPs) has been verified for most proteins identified from honey. These functions are guaranteed, as variable food sources resulted in equal honey protein quality and quantity. Nevertheless, it is clear that several non-discovered nutritive and non-nutritive functions remain elusive and have to be investigated in future studies.

## 5. Conclusions

In conclusion, honey-processing worker bees seem not to adjust their honey protein quality and quantity depending on nectar quality or protein (pollen, bee bread) availability. Those workers add gland-produced proteins as a nutritive protein (nitrogen) source to keep antimicrobial effects constant to preserve hive products, or secreted proteins serve as molecules involved in social immunity (as recently shown for MRJP3 and RNA uptake [42]). Further non-nutritive functions, especially for MRJPs (many of them still with unknown function [31]), might be essential for the biological properties of honey. One of the major functions of bee-secreted peptides and proteins is the prevention of the microbial spoilage of royal jelly, honey and bee bread. These hive products are essential for feeding the brood and the queen, and as a consequence, the survival of the whole colony relies on the quality of the food sources.

## Figures and Tables

**Table 1:** Proteins identified for four different honey-like products (based on feeding sugar solution plus several supplements) using mass spectrometry. Shown are results after analysis with Scaffold_4.8.9 and a newly generated *Apis mellifera* reference protein database [30] (basic settings: minimum number of peptides: 2, protein threshold: 99%, peptide threshold: 95%).

Accession Number	Description	MW	Quantitative Data (Normalized to Total Spectra)			
(NCBI)	(All *Apis mellifera*)	(kDa)	Apiinvert	Sucrose (S)	S + Pollen	S + Quercetin
NP_001011579.1	Major royal jelly protein 1	48.886	21	9	21	12
NP_001011601.1	Major royal jelly protein 3	61.662	12	5	7	7
NP_001011580.1	Major royal jelly protein 2	51.074	9	7	8	5
NP_001011574.1	Glucose oxidase	67.938	6	1	6	3
NP_001011608.1	Alpha-glucosidase Hbg3 precursor	65.565	4	3	5	3
NP_001011599.1	Major royal jelly protein 5	70.236	2	0	2	1
NP_001014429.1	Major royal jelly protein 7	50.541	1	0	1	1

## Supplementary Materials

The following are available online at https://www.mdpi.com/2075-4450/10/9/282/s1, Figure S1: 10% SDS PA gel (colloidal Coomassie stained) showing protein profiles of different monofloral and honeydew honeys, Figure S2: Worker honey bees on a honey comb, in a wooden cage, producing and ripening honey based on dyed sucrose solution, Figure S3: GelAnalyzer results comparing raw volumes for the five most common protein bands of each sample (means ± SD, n = 5–6 per treatment group), Figure S4: Twelve percent SDS PA gel showing protein profiles of ripened honey-like products based on four different feeding regimes, Table S1: Potential identities of eight proteins detected in four different types of honey (black locust, buckwheat, sunflower and heather) from Figure S1.
