# Supplementary material for: The Effect of Diet on the Composition and Stability of Proteins Secreted by Honey Bees in Honey
Source: Insects. 2019 Sep 2;10(9):282. doi: 10.3390/insects10090282 (PMC6780080; doi:10.3390/insects10090282)
Supplement: Supplementary file 1 [file insects-10-00282-s001.pdf]

Supplementary material

The effect of diet on the composition and stability of proteins secreted by honey bees in honey

Oleg Lewkowski[1], Carmen I. Mureşan[1,2], Dirk Dobritzsch[3,4], Matthew Fuszard[3,5], Silvio Erler[1]

[1]Institute für Biologie, Molekulare Ökologie, Martin-Luther-Universität Halle-Wittenberg, Hoher Weg 8, 06120 Halle (Saale), Germany
[2]Institutul de Științele Vieții ”Regele Mihai I al României”, Nutriție moleculară (Genomică și Proteomică), Universitatea de Ştiinţe Agricole sj Medicină Veterinară, Calea Mănăştur 3-5, 400372 Cluj-Napoca, Romania
[3]Proteinzentrum Charles Tanford, Core Facility - Proteomic Mass Spectrometry, Martin-Luther-Universität Halle-Wittenberg, Kurt-Mothes-Straße 3a, 06120 Halle (Saale), Germany
[4]Institut für Biochemie und Biotechnologie, Pflanzenbiochemie, Martin-Luther-Universität Halle-Wittenberg, Kurt-Mothes-Straße 3a, 06120 Halle (Saale), Germany
[5]Zentrum für Medizinische Grundlagenforschung (ZMG), Medizinische Fakultät der Martin-Luther-Universität Halle-Wittenberg, , Ernst-Grube-Str. 40, 06120 Halle (Saale), Germany

Figure S1: 10% SDS PA gel (colloidal Coomassie stained) showing protein profiles of different monofloral and honeydew honeys (BL: black locust - 20 µl, BW: buckwheat - 5 µl, CN: chestnut - 10 µl, HD: forest honey (honeydew) - 10 µl, L: linden - 10 µl, RS: rapeseed - 15 µl, SF: sunflower - 20 µl, WF: white fir (honeydew) - 20 µl, H: heather - 5 µl), diluted 1:10 with sterile water. Different volumes per sample (up to 20 µl) loaded on gel guaranteed equal representation of each honey sample, following preliminary tests and determined protein concentrations. Numbers (1-8) mark characteristic bands, cut out and sent to mass spectrometry (MALDI-TOF/TOF-MS and ESI-QTOF-MS/MS) for identification, following the protocol of Pamminger *et al*. (2016). SwissProt (SIB Swiss Institute of Bioinformatics, Switzerland), NCBI (National Center for Biotechnology Information, USA) and recently published buckwheat (*Fagopyrum esculentum*) and sunflower (*Helianthus annuus*) genomes (Badouin *et al*. 2017, Yasui *et al*. 2016), with help of search engines Mascot (Matrix Science Inc,, USA) and ProteinLynx Global SERVER^{TM} (Waters Corporation, USA), were used for peptide/protein identification. Lack of sequenced plant genomes entered in public databases makes plant protein identification challenging, if not nearly impossible, in some cases (band no. 8). Results on protein identification are summarized in Table S1.

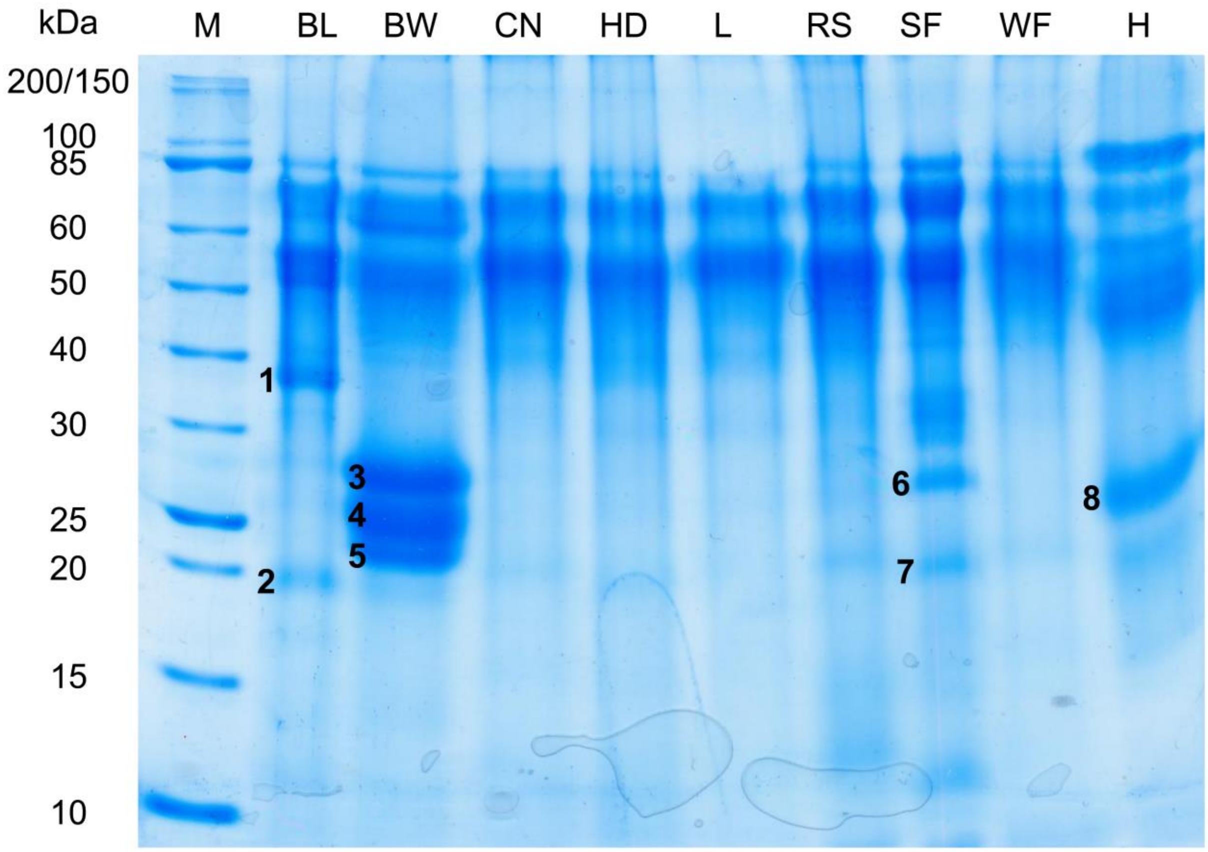

M: marker - Unstained Protein Standard, Broad Range (10-200 kDa) (New England Biolabs, USA)

Table S1: Potential identities of 8 proteins detected in 4 different types of honey (black locust, buckwheat, sunflower and heather) from Figure S1. Shown are results for the first three best matching proteins identified using Mascot, NCBI and published plant genomes (Badouin *et al*. 2017, Yasui *et al*. 2016).

Band no.	Accession Numbers	Description of Proteins	Protein Scores	Matched Peptides	Coverage (%)	Mass (kDa)
**1**	NP_001011579.1	major royal jelly protein 1 precursor [*Apis mellifera*]	5869.59	27	61.34	49.342
	XP_397512.1	PREDICTED: uncharacterized protein LOC408608 [*Apis mellifera*]	3924.41	5	33.15	19.491
	NP_001011580.1	major royal jelly protein 2 precursor [*Apis mellifera*]	2127.81	19	51.55	51.473
**2**	XP_397512.1	PREDICTED: uncharacterized protein LOC408608 [*Apis mellifera*]	3318.74	10	39.78	19.491
	NP_001011579.1	major royal jelly protein 1 precursor [*Apis mellifera*]	824.70	17	34.72	49.342
	NP_001011580.1	major royal jelly protein 2 precursor [*Apis mellifera*]	309.45	10	27.65	51.473
**3**	Fes_sc0031416.1.g000001.aua.1	FES_r1.0.pep [*Fagopyrum esculentum*]	11637.75	8	75.00	16.622
	Fes_sc0012938.1.g000003.aua.1	FES_r1.0.pep [*Fagopyrum esculentum*]	4959.86	5	44.50	23.771
	Fes_sc0042294.1.g000001.aua.1	FES_r1.0.pep [*Fagopyrum esculentum*]	2222.89	5	48.18	23.697
**4**	Fes_sc0078924.1.g000001.aua.1	FES_r1.0.pep [*Fagopyrum esculentum*]	8770.84	9	89.26	15.638
	Fes_sc0035903.1.g000001.aua.1	FES_r1.0.pep [*Fagopyrum esculentum*]	8297.05	8	82.89	16.040
	Fes_sc0000001.1.g000191.aua.1	FES_r1.0.pep [*Fagopyrum esculentum*]	5834.86	8	44.70	23.609
**5**	Fes_sc0035903.1.g000001.aua.1	FES_r1.0.pep [*Fagopyrum esculentum*]	17612.65	9	87.50	16.040
	Fes_sc0078924.1.g000001.aua.1	FES_r1.0.pep [*Fagopyrum esculentum*]	12519.61	6	73.15	15.638
	Fes_sc0000001.1.g000191.aua.1	FES_r1.0.pep [*Fagopyrum esculentum*]	5996.85	12	48.85	23.609
**6**	XP_021981304.1	uncharacterized protein LOC110877465 [*Helianthus annuus*]	6614.21	17	67.08	27.792
	XP_022033614.1	uncharacterized protein LOC110935539 [*Helianthus annuus*]	6407.79	15	60.00	29.237
	XP_022001043.1	uncharacterized protein LOC110898557 [*Helianthus annuus*]	5648.73	16	63.53	29.349
**7**	XP_022018139.1	anther-specific protein SF2 [*Helianthus annuus*]	1760.24	3	28.10	13.556
	XP_397512.1	PREDICTED: uncharacterized protein LOC408608 [*Apis mellifera*]	2370.51	2	19.34	19.491
**8**	not identifiable					

Figure S2: Worker honey bees on honey comb, in a wooden cage, producing and ripening honey-like products based on dyed sucrose (50%) solution. Food was dyed to follow honey production and to facilitate sampling from single cells, reducing risk of cross-contamination.

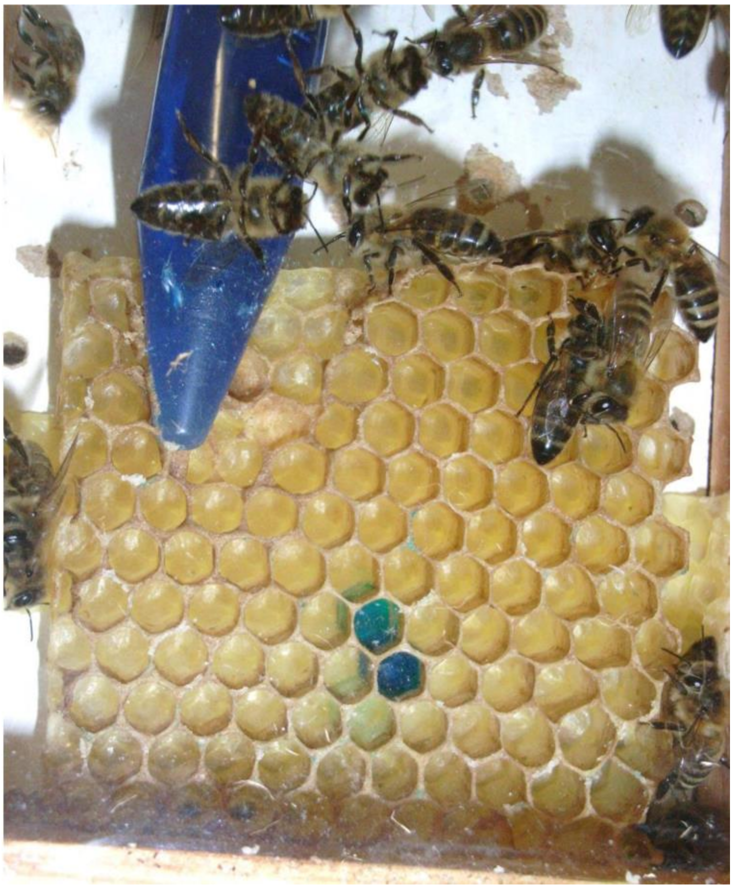

Figure S3: GelAnalyzer results comparing raw volumes for the 5 most common protein bands of each sample (means ± SD, n = 5-6 per treatment group). To account for natural variance in total protein amount, 4 of the 5 protein bands are given as relative values, normalized to the band with highest density (always the band at 50-60 kDa, Figure 1, 2).

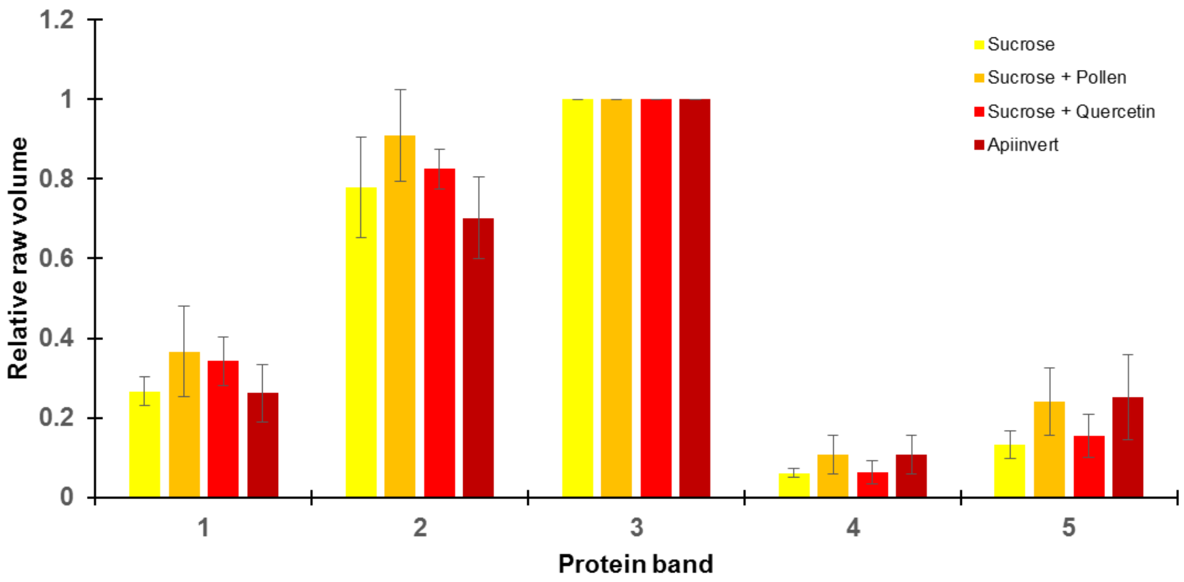

Figure S4: 12% SDS PA gel showing protein profiles of honey-like products based on 4 different feeding regimes (S: sucrose only, SP: sucrose + pollen, SQ: sucrose + quercetin, A: Apiinvert only; numbers refer to different samples/cells of the honey combs). Royal jelly protein extract (RJ) was used as control, as most proteins detected in honey are major royal jelly proteins. (M: marker; Unstained Protein Standard, Broad Range (10-200 kDa) (New England Biolabs, USA))

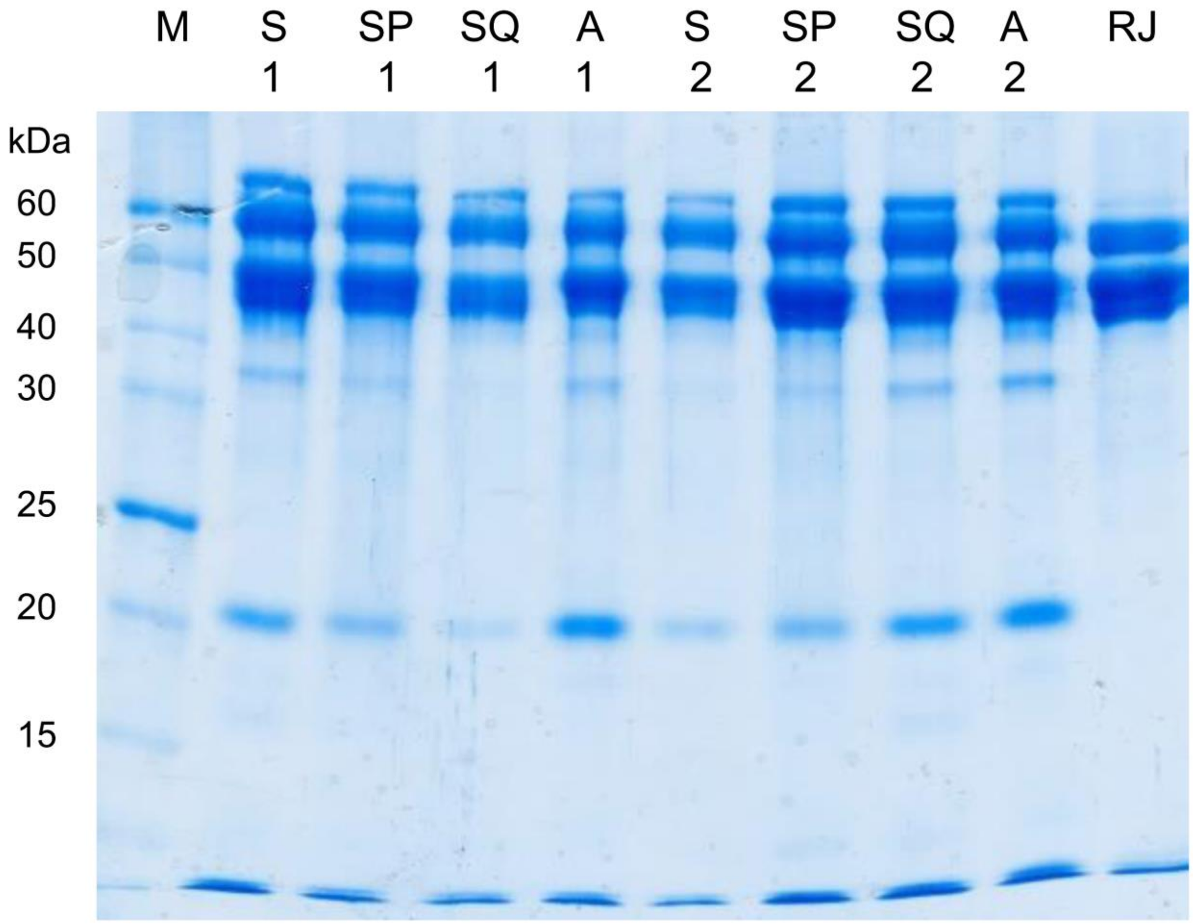

References

Badouin H, Gouzy J, Grassa CJ, Murat F, Staton SE, Cottret L, Lelandais-Brière C, Owens GL, Carrère S, Mayjonade B, *et al*. (2017) The sunflower genome provides insights into oil metabolism, flowering and Asterid evolution. *Nature* 546: 148-152. doi:10.1038/nature22380

Pamminger T, Buttstedt A, Norman V, Schierhorn A, Botías C, Jones JC, Basley K, Hughes WHO (2016) The effects of juvenile hormone on *Lasius niger* reproduction. *J. Insect. Physiol.* 95: 1-7. doi:10.1016/j.jinsphys.2016.09.004

Yasui Y, Hirakawa H, Ueno M, Matsui K, Katsube-Tanaka T, Yang SJ, Aii J, Sato S, Mori M (2016) Assembly of the draft genome of buckwheat and its applications in identifying agronomically useful genes. *DNA Res.* 23: 215-24. doi:10.1093/dnares/dsw012
